# Mitofusin 2 plays a role in oocyte and follicle development, and is required to maintain ovarian follicular reserve during reproductive aging

**DOI:** 10.18632/aging.102024

**Published:** 2019-06-16

**Authors:** Man Zhang, Muhammed Burak Bener, Zongliang Jiang, Tianren Wang, Ecem Esencan, Richard Scott, Tamas Horvath, Emre Seli

**Affiliations:** 1 Department of Obstetrics, Gynecology and Reproductive Sciences, Yale School of Medicine, New Haven, CT 06510, USA; 2 Department of Comparative Medicine, Yale School of Medicine, New Haven, CT 06520, USA; 3 Current address: AgCenter, School of Animal Sciences, Louisiana State University, Baton Rouge, LA 70803, USA; 4 Current address: Foundation for Embryonic Competence, Basking Ridge, NJ 07920, USA

**Keywords:** mitochondrial fusion, follicle maturation, female fertility, ovarian function, ovarian aging

## Abstract

Mitochondria change their shape through fusion and fission in order to adapt to their metabolic milieu. Mitofusin-2 (MFN2) is a key regulatory protein in this process, mediating mitochondrial fusion and interaction with endoplasmic reticulum. Targeted deletion of *Mfn2* in oocytes resulted in mitochondrial dysfunction and female subfertility associated with impaired oocyte maturation and follicle development. Oocytes lacking MFN2 showed shortened telomeres and increased apoptosis, resulting in compromised oocyte quality and accelerated follicular depletion, consistent with a reproductive aging phenotype.

## Introduction

Mitochondria are double membrane-bound, motile organelles that play a central role in several key cellular processes, including energy production, calcium homeostasis, and regulation of apoptosis [1]. Mitochondria have the ability to adapt their size and shape through fusion and fission in response to changes in their metabolic milieu [2]. Mitochondrial fusion and fission also play a critical role in maintaining functional mitochondria when cells experience metabolic or environmental stress [3]. Fusion enables mixing the contents of two mitochondria and helps mitigate stress-related mitochondrial dysfunction through complementation [3]. Fission is primarily used to create new mitochondria. In addition, fission acts as a quality control mechanism by enabling the removal of damaged mitochondrial components and can facilitate apoptosis at times of increased cellular stress. During mouse and human oocyte and early embryo development, mitochondria structure, shape, and number are tightly controlled, suggesting an important role for these processes in reproduction [4].

Mitofusin-1 (MFN1) and mitofusin-2 (MFN2) are GTPases embedded in the outer mitochondrial membrane and are essential for mitochondrial fusion. In addition to the mitofusins, optic atrophy 1 (OPA1) regulates inner mitochondrial membrane fusion, and dynamin related protein 1 (DRP1) is responsible for mitochondrial fission [5,6]. Importantly, mitochondria are in constant interaction with endoplasmic reticulum (ER) [7], and this interaction helps regulate autophagosome formation, mitochondrial fission, Ca^2+^ homeostasis, and apoptosis [8,9]. Studies in mouse embryonic fibroblasts have demonstrated that MFN2 is localized to the mitochondria–ER interface, where, in addition to its role in mitochondrial fusion, it functions to anchor mitochondria to the ER [10]. *MFN2* mutations have been detected in two neurological disorders: Charcot-Marie-Tooth neuropathy type 2A [11] and Axonal Neuropathy with Optic Atrophy [12].

In mice, global germline knockout of *Mfn1, Mfn2*, *Opa1*, and *Drp1* are embryonic lethal [13–15]. Therefore, investigation of the role of these genes in reproduction requires conditional knockout and knockdown approaches. Oocyte-speciﬁc knockout of mitochondrial ﬁssion factor *Drp1* results in female infertility, impaired folliculogenesis, and ovulation [16]. Knockdown of mitochondrial fusion gene *Mfn*2 by siRNA in immature oocytes results in a decline in oocyte maturation and fertilization [17] and lower expression of *Mfn2* is found in cisplatin-induced premature ovarian failure in mice [18]. However, an appreciation of the contribution of mitochondrial fusion genes to adaptive responses of the reproductive system function and aging remains to be elucidated.

In the current study, we investigated the role of MFN2 in female fertility and ovarian function. We found that oocyte-specific targeted deletion of *Mfn2* results in impaired mitochondrial function and dynamics, leading to subfertility associated with impaired follicle development and oocyte maturation. Importantly, absence of MFN2 in the oocyte causes increased apoptosis and shortened telomere length, which results in compromised oocyte quality and follicular depletion, and a phenotype consistent with accelerated reproductive aging.

## RESULTS

### Loss of MFN2 in oocytes results in female subfertility associated with impaired oocyte maturation and embryo development

**Figure 1:**
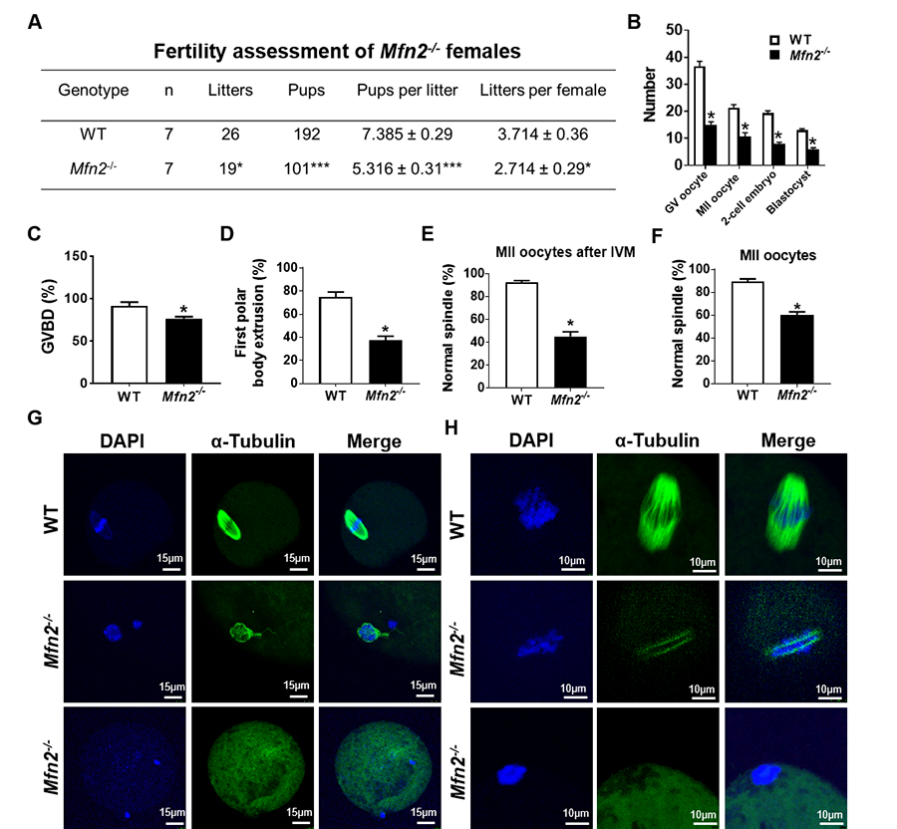
**Subfertility, and impaired follicle, oocyte and embryo development in *Mfn2*^-/-^ mice.** (**A**) Fertility of female *Mfn2^-/-^* (oocyte-specific *Mfn2* knockout, *Mfn2*^fl/fl^/*Zp3-Cre*, referred to as *Mfn2^-/-^*) and WT mice (8-week-old, n = 7 for each genotype) was assessed by mating with WT males of proven fertility (male/female; 1:2) for 12 weeks. *Mfn2^-/-^* mice had smaller litter size (pups per litter) and litters per female compared with WT females. (**B**) Number of GV oocytes, MII oocytes, 2-cell embryos and blastocysts in *Mfn2^-/-^* and WT mice. (**C, D**) Oocytes at GV stage were collected from PMSG-primed *Mfn2^-/-^* and WT mice and analyzed after 18 h of culture under in vitro maturation conditions. Percentages of GVBD and of first polar body extrusion in *Mfn2^-/-^* and WT oocytes are shown. (**E, G**) After 18 h of IVM, *Mfn2^-/-^* and WT MII oocytes were stained with α-tubulin and DAPI. Left column, DAPI (blue); middle column, anti-α-tubulin antibody (green); right column, merged images of DAPI and anti-α-tubulin staining. Percentages of normal spindle morphology in *Mfn2^-/-^* and WT MII oocytes after IVM are shown. (**F, H**) Mature (MII) oocytes were collected from the oviducts of superovulated 8-week-old *Mfn2^-/-^* and WT mice and stained with α-tubulin and DAPI. Percentages of normal spindle morphology in *Mfn2^-/-^* and WT MII oocytes are shown. Data presented as mean ± SEM. *p < 0.05, ***p < 0.0001, vs. WT using *t*-test.

We generated oocyte-specific *Mfn2* knockout mice (*Mfn2*^fl/fl^/*Zp3-Cre*, referred to as *Mfn2^-/-^* throughout the manuscript for simplicity) by crossing floxed *Mfn2* mice to transgenic mice expressing *Zp3-Cre* [19,20], and bred sexually mature female mice (8-week-old, n=7 for each genotype) with WT male mice (12-week-old) of proven fertility (male: female; 1:2). During 12 weeks period of continuous mating, *Mfn2^-/-^* mice consistently exhibited reduced fertility compared to WT with a significant decrease in litter size (5.3 ± 0.2 vs. 7.3 ± 0.3 pups per litter, *p*<0.001) and number of litters per female (2.7 ± 0.29 vs. 3.7 ± 0.36, *p*<0.05) (Figure 1A). Both genotypes exhibited normal mating behavior and vaginal plug formation.

Ability to generate oocytes (germinal vesicle [GV] and metaphase II [MII]), 2-cell embryos, and blastocysts was assessed after injection with PMSG (5IU) or PMSG and hCG (5IU) and mating with WT males as indicated. *Mfn2^-/-^* mice generated significantly lower number of GV oocytes (15 ±1.1 vs 36.6 ±1.8, *p*<0.01), MII oocytes (10.6 ± 1.4 vs 21.3 ± 1.2, *p*<0.01), 2-cell embryos (8 ± 0.5 vs 19.3 ± 0.8, *p*<0.001), and blastocysts (6 ± 0.5 vs 13 ± 0.5, *p*<0.01), compared to WT (Figure 1B).

To characterize the mechanism of impaired oocyte maturation in *Mfn2^-/-^* mice, we performed *in vitro* maturation (IVM) experiments and assessed germinal vesicle breakdown (GVBD, which marks metaphase I), polar body extrusion (which marks metaphase II), and chromatin alignment and spindle morphology. After 18 h of *in vitro* culture, *Mfn2^-/-^* GV stage oocytes had a lower rate of GVBD (76.2 ± 2.3 vs 91.4 ± 4.4%, *p*<0.05) (Figure 1C), lower rate of first polar body extrusion (37.4 ± 3.3 vs 74.6 ± 4.5%, *p*<0.01) (Figure 1D), and significantly lower proportion of normal spindles (45.03 ± 4.1 vs 92.45 ± 1.5%, *p*<0.001) (Figure 1E and 1G). We also tested spindle morphology of *in vivo* generated mature (MII) oocytes collected from the oviduct and found that a significantly lower number of *Mfn2^-/-^* oocytes displayed normal spindle morphology compared to WT (60 ± 2.8, vs 89.3 ± 2.3%, *p*<0.01) (Figure 1F and 1H).

### Targeted deletion of *Mfn2* in oocytes results in mitochondrial dysfunction

**Figure 2:**
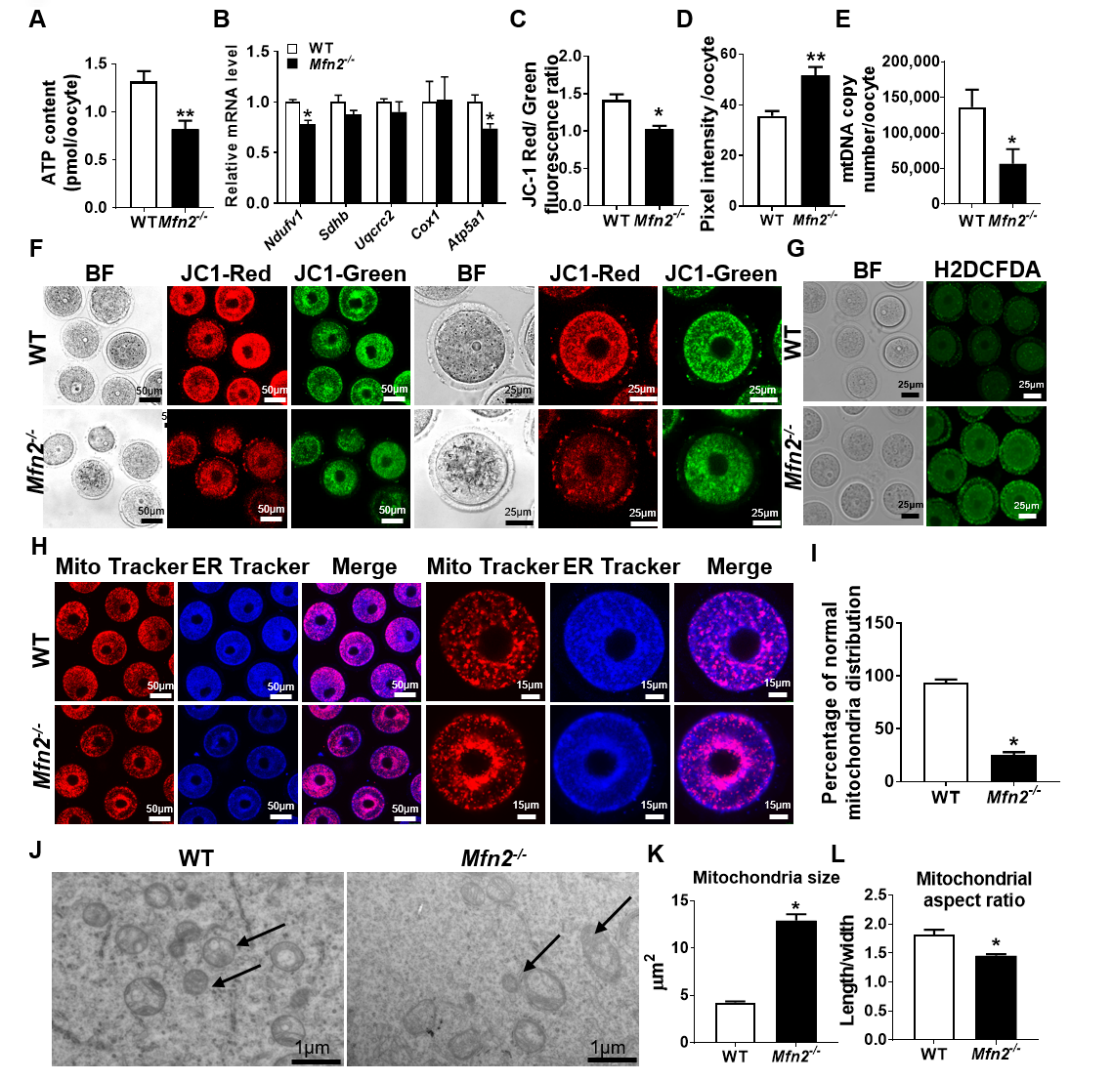
**Mitochondrial function is impaired in *Mfn2^-/-^* oocytes.** (**A**) ATP measurement in *Mfn2^-/-^* and WT mice GV stage oocytes. (**B**) mRNA expression of respiratory chain genes was assessed using qRT-PCR in GV stage oocytes collected from *Mfn2^-/-^* and WT mice. (**C, F**) Representative fluorescent micrographs of GV stage oocytes stained by mitochondria JC-1. Red fluorescence represents J-aggregate while green fluorescence represents JC-1 monomer. Mitochondrial membrane potential is indicated by the red/green fluorescence intensity ratio. (**D, G**) Fluorescence intensity of Carboxy-H_2_DCFDA was used to measure ROS levels after treatment with H_2_O_2_. (**E**) mtDNA copy number was determined by qRT-PCR in GV stage oocytes collected from *Mfn2^-/-^* and WT mice. (**H**) Mitochondria and ER were labeled by immunostaining with MitoTracker (red) and ER-Tracker (blue). (**I**) The percentages of oocytes with normal distribution of mitochondria in the *Mfn2^-/-^* and WT mice. (J) Representative electron microscopic graphs of oocytes from 8-week-old *Mfn2^-/-^* and WT mice (n=3 ovary from different mice assessed in each group). Arrows show mitochondria. (**K, L**) Mitochondrial size and aspect ratio in *Mfn2^-/-^* and WT oocytes. Data presented as mean ± SEM. *p < 0.05, **p < 0.01 vs. WT from *t*-test. ATP: Adenosine triphosphate. *Ndufv1*: NADH dehydrogenase (ubiquinone) flavoprotein 1; *Sdhb*: succinate dehydrogenase complex iron sulfur subunit B; *Uqcrc2*: ubiquinol cytochrome c reductase core protein 2; *Cox1*: cytochrome c oxidase subunit I; *Atp5a1*: ATP synthase, H^+^ transporting, mitochondrial F1 complex, alpha subunit 1.

We then characterized mitochondrial function in GV stage *Mfn2^-/-^* oocytes. *Mfn2^-/-^* oocytes had lower levels of ATP (0.82 ± 0.08 vs 1.31 ± 0.1 pmol, *p*<0.01) (Figure 2A), and expressed decreased amounts of mRNAs coding for electron transport chain (ETC) complex I (*Ndufv1*) and V (*Atp5a1*) proteins (Figure 2B). In addition, *Mfn2^-/-^* oocytes had significantly decreased membrane potential (1.02 ± 0.04 vs 1.41 ± 0.07, *p*<0.01) (Figure 2C and 2F), and elevated levels of ROS compared to WT (51.72 ± 3.2 vs 35.69 ± 1.9, pixel intensity, *p* < 0.01) (Figure 2D and 2G). We also found that the mtDNA copy number were significantly lower (56,655 ± 20,659 vs 136,268 ± 24,588, *p*<0.05) in *Mfn2^-/-^* oocytes (Figure 2E).

When we assessed mitochondria and ER distribution in *Mfn2^-/-^* GV oocytes, we found increased perinuclear aggregation compared to WT (25 ± 2.8 vs 94.33 ± 2.3, *p*<0.01) (Figure 2H and 2I); a distribution that was reported to be associated with diminished ATP generation capacity [21]. Electron microscopy (EM) showed mitochondria to be larger (12.91 ± 0.67 vs 4.25 ± 0.12 μm^2^, *p*<0.001) and with smaller aspect ratio (length/width) (1.45 ± 0.02 vs 1.82 ± 0.08, *p*<0.01) in *Mfn2^-/-^* oocytes compared to WT (Figure 2J, 2K and 2L). Collectively, these data suggested that mitochondrial function and dynamics are impaired in *Mfn2^-/-^* oocytes.

### Gene expression is altered in *Mfn2^-/-^* oocytes

**Figure 3:**
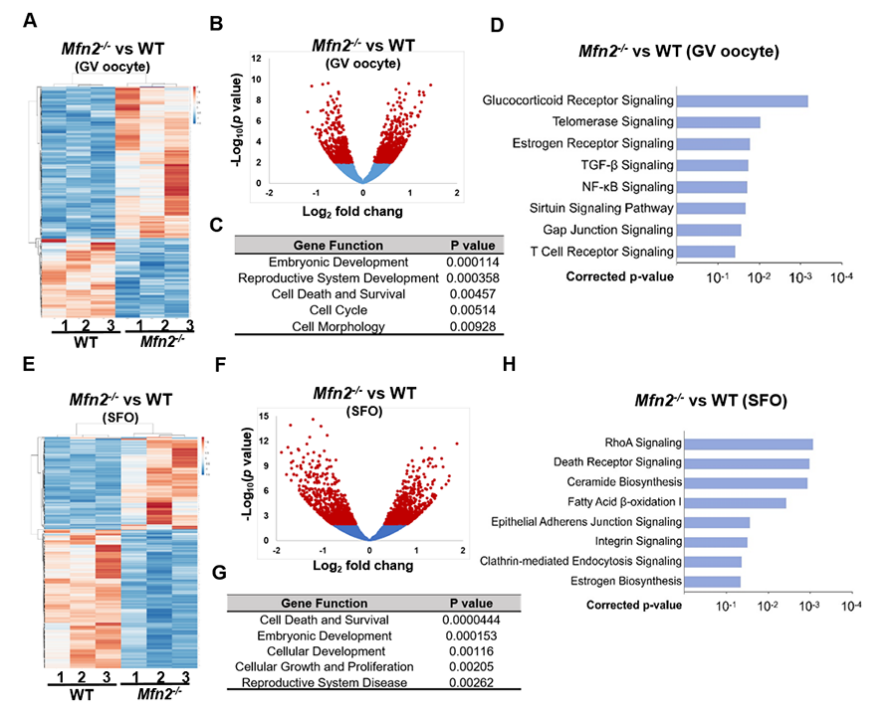
**Gene expression is altered in *Mfn2^-/-^* GV stage and secondary follicle enclosed oocytes.** (**A, E**) Heatmaps showing differentially expressed genes in *Mfn2^-/-^* and WT GV oocytes and secondary follicle enclosed oocytes (SFO) from 8-week-old mice. The color spectrum ranging from red color to blue color indicates normalized levels of gene expression from high to low. (**B, F**) Volcano plots for RNA-seq comparing *Mfn2^-/-^* and WT GV oocytes and SFOs. Red spot represents –log_10_ (*p*-value) ≥ 2; blue spot represents the – log_10_ (*p*-value) < 2. (**C, G**) Gene ontology (GO) cluster analysis of the significant over-representation of elements in *Mfn2^-/-^* and WT GV oocytes and SFOs from 8-week-old mice. (**D, H**) Pathway enrichment analysis in *Mfn2^-/-^* oocytes compared to WT GV oocytes and SFOs.

To delineate the genes and pathways affected in MFN2-deficient oocytes, RNA sequencing (RNAseq) analysis was performed in GV oocytes and secondary follicle enclosed oocytes (SFOs) derived from *Mfn2^-/-^* and WT mice. Hierarchical clustering of the differentially expressed genes partitioned into two distinct clusters to separate *Mfn2^-/-^* and WT GV oocytes (Figure 3A). A total of 363 genes were significantly differentially expressed (*p*<0.05) in *Mfn2^-/-^* compared to WT GV oocytes, with 241 up-regulated and 122 down-regulated genes (Figure 3B). Gene ontology (GO) analysis indicated significant over-representation of elements involved in regulation of embryonic development, cell death and survival, and cell morphology (Figure 3C). Most significantly regulated pathways in *Mfn2^-/-^* GV oocytes were listed in Figure 3D. Notably, telomerase signaling pathway and sirtuin signaling pathway were affected (Figure 3D).

Hierarchical clustering of the differentially expressed genes also partitioned into two distinct clusters to separate *Mfn2^-/-^* and WT SFOs (Figure 3E). A total of 1,041 genes were significantly differentially expressed (*p*<0.05) in *Mfn2^-/-^* SFOs compared to WT, with 510 up-regulated and 531 down-regulated (Figure 3F). The biological processes significantly represented at this comparison included regulation of cell death and survival, cellular development and cellular growth (Figure 3G). Pathway analysis revealed essential regulated signaling pathways as listed in Figure 3H. Importantly, death receptor signaling pathway, ceramide biosynthesis and adherens junction signaling pathway, were all affected in *Mfn2^-/-^* SFOs (Figure 3H).

### Targeted deletion of *Mfn2* in oocyte results in accelerated depletion of ovarian follicular reserve

Our RNAseq analysis showed that aging-related telomerase and sirtuin signaling pathways were affected, and genes involved in cell death and survival were differentially expressed in *Mfn2^-/-^* oocytes. We had also found accelerated follicle depletion in mice deficient for mitochondrial stress response gene *Clpp* [22]. We therefore next characterized how the follicle pool changes throughout mouse reproductive lifespan in unstimulated *Mfn2^-/-^* and WT mice ovaries.

**Figure 4:**
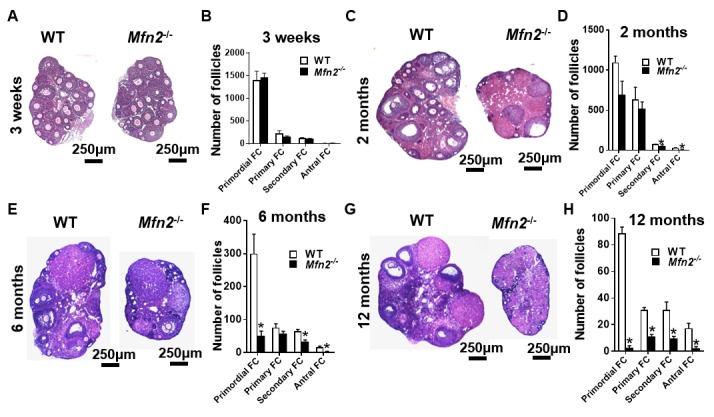
**Accelerated follicle depletion in *Mfn2^-/-^* mice.** (**A, C, E, G**) Representative micrographs of 3-weeks and 2-, 6- and 12-months old *Mfn2^-/-^* and WT mice ovary sections stained with hematoxylin and eosin. (**B, D, F, H**) Follicle counts in 3-weeks and 2-, 6- and 12-months old *Mfn2^-/-^* and WT mice ovaries. Data presented as mean ± SEM. *p < 0.05 vs. WT using *t*-test.

Pre-pubertal (3-week-old) *Mfn2^-/-^* and WT mice had similar number of follicles at different stages of development (Figure 4A and 4B). At 2 months, the number of primordial (which represent ovarian follicular reserve) and primary follicles did not differ between *Mfn2^-/-^* and WT ovaries, while *Mfn2^-/-^* ovaries had decreased number of secondary and antral follicles (Figure 4C and 4D). By 6 months, *Mfn2^-/-^* ovaries had significantly lower number of primordial as well as secondary and antral follicles (Figure 4E and 4F). At 12 months (Figure 4G and 4H), *Mfn2^-/-^* ovaries showed dramatically decreased number of follicles at all stages.

We also assessed serum levels of hormones associated with ovarian reserve. FSH levels were increased in 2-, 6-, and 12-month-old *Mfn2^-/-^* mice compared to wild type (Supplementary Figure 1A), and serum AMH levels were significantly lower in 2-, 6-, and 12-month-old *Mfn2^-/-^* mice compared to WT (Supplementary Figure 1B). In addition, the ovaries of *Mfn2^-/-^* mice were significantly smaller in size (1.7 ± 0.1 vs 3.5 ± 0.1 mm^2^ at 8-week, *p*<0.001; n = 5 for each genotype) (Supplementary Figure 2A and 2B).

These results demonstrate that oocyte-specific deletion of *Mfn2* causes accelerated follicular depletion, and results in a phenotype similar to that observed in women with diminished ovarian reserve and in those approaching peri-menopause.

### *Mfn2^-/-^* secondary follicle-enclosed oocytes show increased apoptosis associated with elevated levels of ceramide and decreased expression of E-cadherin and Connexin 37

We next sought to investigate the potential mechanisms of accelerated follicle loss in *Mfn2^-/-^* mice. Oocyte apoptosis results in follicular developmental arrest and follicular atresia [23], and impaired oocyte-granulosa cell communication may lead to oocyte apoptosis [24]. Our bioinformatics analysis revealed that apoptosis, ceramide biosynthesis, and junction signaling pathways were differentially regulated in *Mfn2^-/-^* SFOs (Figure 3H). We therefore tested whether apoptosis is increased in *Mfn2^-/-^* oocytes, and whether pathways implicated in apoptosis-induction (ceramide biosynthesis) and oocyte-granulosa cell communication (junction signaling pathway) are indeed affected.

**Figure 5:**
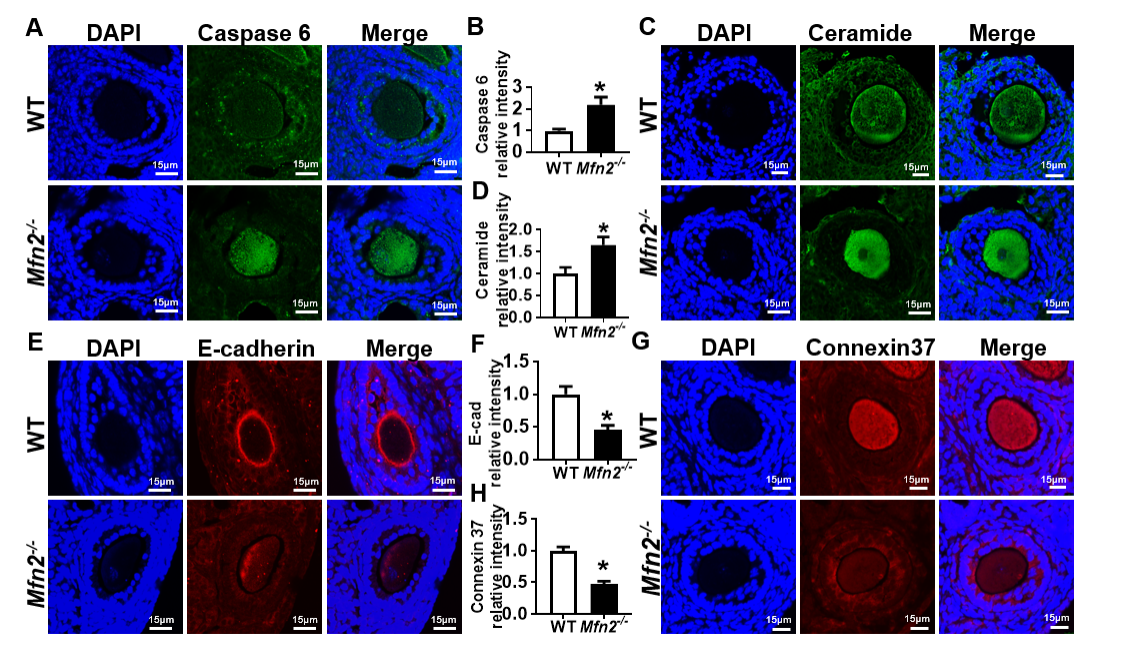
**Increased apoptosis in *Mfn2^-/-^* secondary follicle-enclosed oocytes is associated with increased ceramide and decreased junction protein expression.** (**A, C**) Immunofluorescence staining of caspase-6 (green) and ceramide (green) in secondary follicles of *Mfn2^-/-^* and WT mice ovaries. DAPI was used to stain nuclei (blue). (**B, D**) Quantitative analysis of caspase-6 and ceramide immunofluorescence in secondary follicles of *Mfn2*^-/-^ and WT mice ovaries. (**E, G**) Immunofluorescence staining of E-cadherin (red) and Connexin37 (red) in secondary follicles of *Mfn2^-/-^* and WT mice ovaries. (**F, H**) Quantitative analysis of E-cadherin and Connexin37 immunofluorescence in secondary follicles of *Mfn2^-/-^* and WT mice ovaries. Data presented as mean ± SEM. *p < 0.05 vs. WT using *t*-test.

We found increased expression of apoptosis effector protein caspase 6 in *Mfn2^-/-^* SFOs compared to WT (2.19 ± 0.34 vs 1 ± 0.08, *p*< 0.05) (Figure 5A and 5B). We also tested ceramide levels in *Mfn2^-/-^* SFOs, as increased ceramide is reported to induce apoptosis by activating effector caspases [25]. We found ceramide levels in *Mfn2^-/-^* SFOs to be significantly higher (1.65 ± 0.18 vs 1 ± 0.14, *p*< 0.05) compared to WT (Figure 5C and 5D).

We next assessed the expression of E-cadherin and Connexin 37 (Cx37), key factors in oocyte-granulosa cell communication involved in adherens and gap junction formation, respectively. E-cadherin is expressed exclusively in the oocyte and localized to oocyte membrane [26]. Immunofluorescent labeling showed proper localization and expression of E-cadherin in WT secondary follicles while its levels were significantly decreased in *Mfn2^-/-^* (0.45 ± 0.07 vs 1 ± 0.12, *p*< 0.05) (Figure 5E and 5F). Similarly, we found significantly decreased Cx37 protein levels in *Mfn2^-/-^* SFOs (0.47 ± 0.04 vs 1 ± 0.06, *p*< 0.01) (Figure 5G and 5H). Collectively, our findings suggest that increased apoptosis in *Mfn2^-/-^* secondary follicle-enclosed oocytes is associated with increased levels of ceramide in the oocyte and impaired communication between the oocyte and surrounding granulosa cells.

It is noteworthy that transzonal processes (TZPs), which anchor the first layer of granulosa cells to the oocyte to initiate gap junction formation [27], were not significantly different in *Mfn2^-/-^* SFOs and cumulus oocyte complexes (COCs) compared to WT (Supplementary Figure 3A-D). In addition, the expression of *Gdf9* and *Bmp15* were not significantly changed in *Mfn2^-/-^* SFOs and GV oocytes, suggesting that impaired follicular development was not mediated through a decrease in these key oocyte-derived mediators of folliculogenesis (Supplementary Figure 3E-F).

### *Mfn2^-/-^* GV oocytes have shortened telomeres with decreased expression of telomere protective protein TRF1

Mammalian telomeres consist of tandem repeats of the sequence TTAGGG and protect the stability of chromosomes [28]. Most cells lack telomerase, the enzyme responsible for maintenance of telomere length, and their telomeres shorten through consecutive cell divisions [29]. Telomere shortening is a key mechanism leading to cell senescence, and also has been associated with decreased oocyte quality through disruption of chromosome alignment and spindle structure during meiosis [30]. As we uncovered impaired oocyte maturation in *Mfn2^-/-^* mice and our RNAseq analysis showed telomerase signaling pathway to be affected in *Mfn2^-/-^* GV oocytes, we next assessed telomere length in *Mfn2^-/-^* and WT GV oocytes using quantitative real-time PCR [28]. Cumulus cells (CCs) and white blood cells (WBCs) were also assessed as somatic controls.

**Figure 6:**
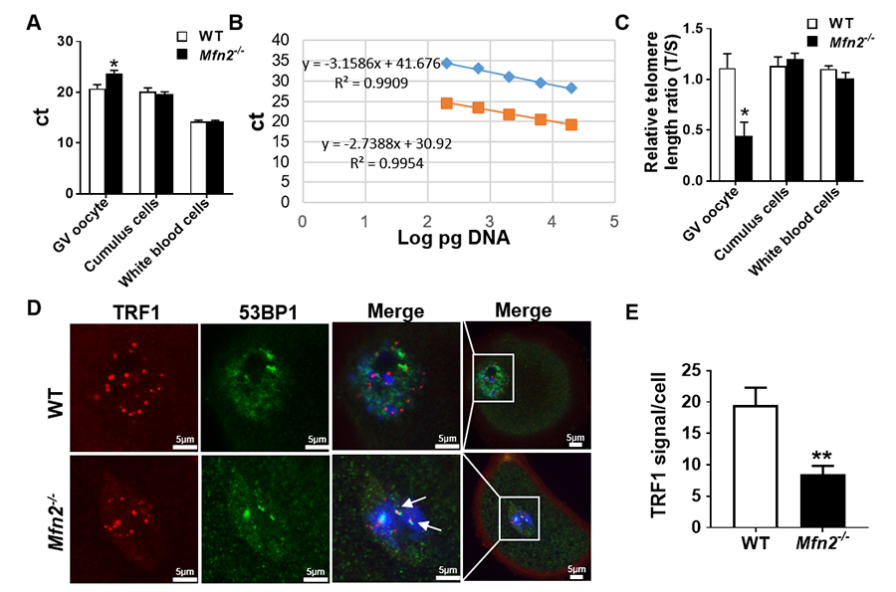
**The telomere is shorter in *Mfn2^-/-^* oocyte.** (**A**) Ct values from quantitative real-time PCR (qRT-PCR) of GV oocytes, cumulus cells and white blood cells form *Mfn2^-/-^* and WT mice. (**B**) Standard curves were generated by serial dilution of known amounts of DNA to calculate relative DNA concentrations (log DNA) from Ct values of the qRT-PCR products. Orange squares, telomeres; blue diamonds, 36B4 single copy gene control. The correlation regression equation and coefficients (R2) of Ct versus log DNA are shown. (**C**) The relative telomere length of GV oocytes, cumulus cells and white blood are represented as ratio of T/S. (**D**) Immunofluorescence double staining of 53BP1 (green) and TRF1 (red) in cumulus oophorus complexes of *Mfn2^-/-^* and WT mice. DAPI was used to stain nuclei (blue). (**E**) Quantitative analysis of TRF1 immunofluorescence in *Mfn2^-/-^* and WT GV oocytes. The arrow showed the co-localization of 53BP1 and TRF1. Data presented as mean ± SEM. **p < 0.01, *p < 0.05 vs. WT using *t*-test.

Telomere length of *Mfn2^-/-^* GV oocytes was significantly shorter compared to WT, as more PCR cycles (23.68 ± 0.63 vs 20.7 ± 0.78, *p*< 0.05) were required to reach the threshold (Figure 6A), and telomere/single-copy gene ratio (T/S), a good indication of relative telomere length [28], was lower (Figure 6B and 6C). There was no difference in the telomere length of CCs and WBCs in *Mfn2^-/-^* mice compared to WT (Figure 6C).

Recent studies have shown an important role for telomeric repeat-binding factor 1 (TRF1), a molecule of shelterin protein complex, in protecting the telomere DNA integrity and stability [31]. Loss of TRF1 leads to DNA damage response and accumulation of DNA repair factors such as 53BP1 [32,33]. To investigate whether the shortened telomere in *Mfn2^-/-^* oocytes is associated with decreased protection and/or accelerated telomere damage, we performed co-immunofluorescence staining of TRF1 and 53BP1, and found TRF1 to be significantly decreased in *Mfn2^-/-^* GV oocytes’ nuclei compare to WT (Figure 6D and 6E). In addition, TRF1 co-localized with 53BP1 in *Mfn2^-/-^* oocytes, indicating telomere damage in the *Mfn2^-/-^* oocytes (Figure 6D, shown by arrows).

## DISCUSSION

In the current study, we investigated whether female fertility is impaired in mice with oocyte-specific targeted deletion of *Mfn2*, a key regulator of mitochondrial fusion. We found that *Mfn2*^-/-^ mice produce less pups compared to WT (Figure 1A). They also generate less mature antral follicles (Figure 4) and less mature oocytes and embryos (Figure 1B). Our findings demonstrate that absence of MFN2 in oocytes results in female subfertility associated with impaired folliculogenesis and oogenesis. Consistent with our observations, Liu et al. had previously reported that knockdown of *Mfn*2 by siRNA in immature oocytes results in mitochondrial dysfunction and abnormal spindle formation, and causes a decline in oocyte maturation and fertilization [17]. Importantly, we observed that deletion of *Mfn2*^-/-^ in oocytes results in decreased fertility, but not in a complete loss of reproductive function. MFN2 plays a key role in mitochondrial fusion and mitochondria-ER interactions. Therefore, the limited (but not absent) reproductive potential in *Mfn2*^-/-^ female mice, could be explained by compensatory effects of other proteins, such as MFN1.

We then characterized mitochondrial function, distribution, and dynamics in *Mfn2^-/-^* oocytes, focusing first on parameters related to mitochondrial energy generation. We found *Mfn2^-/-^* oocytes to have lower expression of the ETC genes and decreased ATP production (Figure 2). Therefore, our findings support existing data suggesting an association between decreased oocyte ATP levels and impaired oocyte maturation. Indeed, two peaks of ATP production have been observed during oocyte maturation [34]. The first one is during germinal vesicle breakdown (GVBD) and the second one is during the extrusion of the first polar body. Importantly, the second peak (associated with first polar body extrusion) is absent in oocytes that fail to complete the first meiotic division. Similarly, ATP content of human oocytes have been linked to developmental potential and IVF outcome [35].

Mitochondria are motile organelles and changes in mitochondrial function have been associated with differential cellular distribution of mitochondria [36], with three main mitochondrial distribution patterns described as subplasmalemmal, pan-cytoplasmic and perinuclear [37]. Our results revealed perinuclear aggregation of mitochondria in *Mfn2^-/-^* oocytes (Figure 2H), similar to that described in blastomeres with diminished ATP generating capacity [21]. When we assessed mitochondrial size and shape (dynamics) in *Mfn2^-/-^* oocytes by electron microscopy, we found that *Mfn2^-/-^* mice oocytes have bigger and rounder mitochondria (Figure 2J-L). In addition, we found mtDNA copy number to be lower in *Mfn2^-/-^* oocytes compared to WT (Figure 2E). This finding is in contrast with high mtDNA copy number detected in *Clpp*-deficient mice oocytes with severe metabolic dysfunction and mitochondrial stress [22], and suggest that decreased as well as increased amount of mtDNA may be detected in oocytes with metabolic distress, depending on the pathways involved.

After characterizing the developmental and metabolic changes that occur in MFN2-deficient oocytes, we performed RNAseq analysis in SFOs and GV oocytes from *Mfn2^-/-^* and WT mice to identify genes and pathways affected by MFN2 (Figure 3). In total, we found 1,041 genes to be differentially expressed in *Mfn2^-/-^* SFOs and 363 in *Mfn2^-/-^* GV oocytes compared to WT. Of particular interest, death receptor signaling, ceramide biosynthesis and adherens junction signaling pathways were affected in *Mfn2^-/-^* SFOs (Figure 3H), while aging-related telomerase and sirtuin signaling pathways were affected in *Mfn2^-/-^* GV oocytes (Figure 3D).

The number of resting follicles available in the ovary, also called *ovarian reserve*, is the primary determinant of response to ovarian stimulation in women undergoing infertility treatment [38]. Targeted deletion of *Mfn2* in oocytes resulted in accelerated follicular depletion, similar to that observed in mice with global germline deletion of mitochondrial stress response gene *Clpp*, where oocytes have severe metabolic dysfunction [22,39]. Specifically, we found a dramatically decreased number of primordial follicles starting at 6 months in *Mfn2^-/-^* mice, depletion of primordial follicles and significantly decreased number of primary and secondary follicles at 12 months, and decreased serum AMH levels, consistent with a diminished ovarian reserve phenotype (Figure 4). Our findings regarding accelerated follicular depletion in *Mfn2*^-/-^ mice are supported by a previous study showing decreased MFN2 expression in mice that develop apoptotic oocyte loss and ovarian failure in response to cisplatin treatment [18].

The reproductive phenotype that we observed in this study should be interpreted within the context of the experimental approach used for targeted deletion. Zp3-driven targeted deletion in oocytes occurs only after the follicle reaches the primary follicle stage. Therefore, Zp3-Cre system does not allow targeted deletion in dormant oocytes stored in primordial follicles, making its use challenging within the context of reproductive aging. Alternatively Vasa-Cre allows Cre enzyme to be expressed in oocytes at all developmental stages (including during fetal life and primordial follicles before and after sexual maturation) [40]. However, Vasa-Cre has been found to have leaky expression in the early embryo and may cause lethality [40,41], limiting its use. In the current manuscript, utilization of the Zp3-Cre system allowed reliable assessment of the role of MFN2 in oocytes enclosed in maturing follicles. In addition, it enabled us to conclude that accelerated loss of primordial follicles is caused by accelerated recruitment rather than a direct effect on these follicles (as the Zp3-Cre system is not yet active in primordial follicle-enclosed oocytes).

Pathological or iatrogenic insults that result in oocyte apoptosis may accelerate follicular depletion by inducing arrest of follicle development [22,42]. Indeed, inhibition of apoptotic pathways may prevent chemotherapy-induced follicular atresia [43]. We confirmed increased apoptotic activity in *Mfn2^-/-^* SFOs by demonstrating higher expression of the apoptotic effector caspase 6 (Figure 5A). Among the several mechanisms that may result in oocyte apoptosis, compromised oocyte-granulosa cell communication [24] is particularly noteworthy because RNAseq analysis identified junction signaling pathway as being significantly affected in *Mfn2^-/-^* SFOs. Consistent with RNAseq findings, we found expression of adherens and gap junction proteins to be significantly decreased in *Mfn2^-/-^* SFOs (Figure 5E-H). Ceramide, which induces apoptosis by releasing cytochrome c from mitochondria and activating effector caspases [25], was also elevated in *Mfn2^-/-^* SFOs (Figure 5C), consistent with RNAseq findings. It is noteworthy that ceramide accumulates in aging oocytes, potentially playing a role in age-related acceleration of follicle depletion [44]. Our findings are consistent with previous reports suggesting that mitochondrial fusion plays a protective role against apoptosis [45,46].

Reproductive aging is associated with a decline in oocyte quality in addition to decreased number of follicles in the ovary [47]. Telomere shortening may contribute to impaired reproductive function and reproductive aging as it is associated with increased meiotic defects, spindle and chromosome abnormalities, and embryo fragmentation [30,48]. RNAseq analysis uncovered telomere signaling pathway to be significantly affected in *Mfn2^-/-^* GV oocytes, which had shorter telomeres (Figure 6A-C), and lower expression of telomere protective protein TRF1 (Figure 6D-F), with increased co-localization of 53BP1 with TRF1. These findings are in line with recent studies, demonstrating an essential role for mitochondria in maintaining telomere length during aging [49].

Reactive oxygen species (ROS) generated by mitochondria are considered major contributors to telomeric DNA damage and may have a significant impact on cellular senescence [49]. Increased ROS may cause telomere damage through ineffective DNA repair and increased mtDNA damage [50]. In the current study, in addition to significantly increased ROS generation, we found dramatically decreased mtDNA copy number in *Mfn2^-/-^* oocytes compared to WT (Figure 2). There seems to be a close relationship between mtDNA copy number and telomere length [51,52], and a positive correlation was also observed in a number of disease models [53,54]. Importantly, mtDNA depletion in skeletal myoblasts induces telomere shortening [52]. Our findings suggest that targeted deletion of *Mfn2* in oocytes results in mitochondrial dysfunction, increased ROS, and lower mtDNA content, which in turn is associated with telomere shortening, possibly contributing to subfertility and accelerated follicular depletion.

In this study, we uncovered that oocyte specific deletion of *Mfn2* results in female subfertility due to impaired oocyte maturation and follicular development. In addition, absence of *Mfn2* in the oocyte results in accelerated follicular depletion and a phenotype similar to that observed in women with diminished ovarian reserve. Studies are underway to delineate how intricate interactions between metabolism, mitochondrial function, and reproduction determine reproductive success and failure in women through different stages of reproductive lifespan.

## MATERIALS AND METHODS

### Animals

All animal care and experimental procedures were conducted in accordance with Yale University animal research requirements under the protocols approved by the Institutional Animal Care and Use Committee (protocol # 2017-11207). *Mfn2*^flox/flox^ mice (stock number 026525) and *Zp3*-Cre mice [19,20] (where Cre is driven by Zp3 promoter) in C57BL/6 background (stock number 003651) were purchased from The Jackson Laboratory. *Mfn2*^flox/flox^ mice were crossbred with *Zp3-Cre* mice to produce mice with oocyte-specific *Mfn2* deletion (*Mfn2*^fl/fl^/*Zp3-Cre* mice), and for simplicity, they are referred to as *Mfn2^-/-^* mice hereafter. Genotyping was carried out using the primers shown in Supplementary Table 1.

### Assessment of fertility

To assess the fertility of *Mfn2^-/-^* female mice, seven mating cages were set up. In each cage, one adult male mouse (12-week-old) with proven fertility was placed with two female mice (*Mfn2^-/-^* and WT). Male mice were rotated every week. Fertility test was carried out for 12 consecutive weeks. Cages were monitored daily. Number of pups and litters were recorded.

### Histomorphometric analysis of folliculogenesis in ovaries

Ovaries from 8-week-old *Mfn2^-/-^* and WT female mice were extracted and fixed in 4% (w/v) paraformaldehyde in Dulbecco's phosphate buffered saline (DPBS) at room temperature overnight, and stored at 4°C in fresh 70% ethanol until processed. Ovaries were then dehydrated, embedded in paraffin, sectioned (5 μm), and stained with hematoxylin and eosin (H&E). Every fifth section on the slides were assessed, and only the follicles that contain oocytes with clearly visible nucleus were counted. Primordial, primary, secondary and antral follicles were classified as described previously [55]. Briefly, primordial follicles were defined as an oocyte surrounded by a single layer of squamous granulosa cells. Primary follicles possessed an oocyte surrounded by a single layer of cuboidal granulosa cell layer. Secondary follicles consisted of an oocyte surrounded by two or more layers of cuboidal granulosa cells with no visible antrum. Antral follicles contained four or more layers of granulosa cells with a clearly defined single antral space.

### Follicle, oocyte, and embryo collection

Eight-week-old female *Mfn2^-/-^* and WT mice were used to collect oocytes, embryos, and follicles. To collect germinal vesicle (GV) stage oocytes, 5 IU PMSG (Sigma, St. Louis, MO) was injected intraperitoneally and ovaries were extracted 44-48 h after the injection. To retrieve the oocytes, ovaries were punctured with a 26-gauge needle. Collected oocytes were placed in M2 medium (Sigma, St. Louis, MO) and 10 μM milrinone (Sigma, St. Louis, MO) to prevent meiotic resumption. To obtain mature oocytes, an additional injection of 5 IU of human chorionic gonadotrophin (hCG; Sigma, St. Louis, MO) to induce oocyte maturation and ovulation was given 48 h after the PMSG injection. Unfertilized oocytes at metaphase of the second meiotic division (MII) were collected from oviducts 14 h after the hCG injection. To collect fertilized embryos, females were mated with WT males immediately after the hCG injection. The following morning, mating was confirmed by the presence of a vaginal plug. Two-cell embryos were collected 44 h after hCG injection from the oviducts in KSOM medium (Millipore, Darmstadt, Germany). Blastocysts were collected 92 h after hCG injection from uterus into M2 medium (Sigma, St. Louis, MO). Secondary follicles were collected by digesting the ovaries with 1.5 mg/mL collagenase type V (Sigma, St. Louis, MO) for 1 h at 37°C in M2 medium (Sigma, St. Louis, MO) [16].

### Quantification of ATP

ATP content of individual oocytes was determined using the ATP bioluminescent somatic cell assay kit (Sigma, St. Louis, MO). Oocytes were collected, lysed, and stored individually in 100 μl of ATP releasing reagent at -80ºC. ATP Assay Mix Working Solution (100 μl, 1:25 diluted from ATP Assay Mix Stock Solution) was added individually to 96-well plate wells and kept at room temperature for 5 min. Fifty μl of sample or standard to be assayed weas mixed with 50 μl of ATP releasing reagent and shaken briskly; 100 μl of this mix was transferred individually to the 96-well plate containing 100 μl of ATP Assay Mix Working Solution that was previously prepared and equilibrated, and the amount of light emitted was measured immediately with Dynex MLX microliter plate luminometer (Dynex Technologies, Chantilly, VA). ATP in single oocyte samples was calculated by comparison to a standard curve generated over the range 2.5–500 fmol/100 μl.

### Quantitative reverse-transcription polymerase chain reaction (qRT-PCR)

Total RNA was obtained from oocytes using RNAqueous Microkit (Thermo Fisher Scientific, Waltham, MA) and was treated with DNase I (Thermo Fisher Scientific) for genomic DNA contamination. Reverse transcription was performed using the RETROscript kit (Thermo Fisher Scientific, Waltham, MA) in two steps: first, template RNA and random primers were incubated at 85ºC for 3 min to eliminate any secondary structures, and then the buffer and enzyme were added and the reaction was carried out at 42ºC for 1 h. qRT-PCR was carried out in an iCycler (Bio-Rad Laboratories, Hercules, CA). cDNA was prepared as described above, and assayed in triplicates. Each experiment was repeated at least three times using individual animals from each genotype. Each 10-μl reaction contained 5 μl of SYBR Green supermix (Bio-Rad Laboratories, Hercules, CA), 3 μl of H2O, 0.5 μl of each primer, and 1 μl of cDNA. TaqMan Gene expression assays (Life Technologies, Carlsbad, CA) were also used following manufacturer’s instructions. Briefly, each 20-μl reaction contained 1 μl of 20X TaqMan gene expression assay, 10 μl of 2X TaqMan Gene expression master mix, 4 μl of cDNA template, and 5 μl of H2O. The 2-ΔΔCT (cycle threshold) method was used to calculate relative expression levels after normalization to *Actb* (β-actin) levels. The primers used for real-time PCR reactions were included in Supplementary Table 1.

### Electron microscopic analysis

For transmission electron microscopy, 3 *Mfn2^-/-^* and 3 WT female mice were deeply anesthetized 44 h after PMSG injection followed by perfusion of 4% paraformaldehyde/PBS. Both ovaries were fixed at 4ºC overnight with the fixative solution (paraformaldehyde 2%, glutaraldehyde 2.5% in cacodylate buffer 0.1 M, pH 7.4). After ovaries were rinsed in the same buffer twice, they were postfixed in 1% OsO4 in 0.1 M cacodylate buffer at room temperature for 60 min. Specimens were stained *en bloc* with 2% aqueous uranyl acetate for 30 min, dehydrated in a graded series of ethanol to 100% and embedded in Poly/bed 812 resin. Then the blocks were polymerized in a 60°C oven for 24 h and thin sections (60 nm) were cut by a Leica ultramicrotome and post-stained with 2% uranyl acetate and lead citrate. Cell sections were examined with a FEI Tecnai transmission electron microscope and digital images were recorded with an Olympus Morada CCD camera and iTEM imaging software. Oocytes were imaged at 11,500X magnification. ImageJ software was used to measure the mitochondria area, length and width.

### Analysis of mitochondrial membrane potential

Mitochondrial membrane potential was assessed by incubating oocytes with the mitochondrial membrane dye JC-1 (Invitrogen, Carlsbad, CA) at 2 μg/ml for 30 min at 37°C in the dark. Following the incubation, JC-1-free media was used to wash the oocytes. Then oocytes were imaged by Leica SP5 spectral scanning confocal microscopy in green and red fluorescence channels. For the green channel, excitation was performed at 488 nm and emission at 530 nm. For the red channel, excitation was performed at 568 nm and emission at 590 nm. Quantification of the pixel intensity was assessed by Image J software.

### Determination of ROS levels

Reactive oxygen species (ROS) generation was induced by exposing oocytes to 20 mM H_2_O_2_ for 5 min and then measured by incubating these oocytes with 30 µM H2DCFDA (6-carboxy-2',7’-dichlorodihydrofluorescein diacetate, Life Technologies, Carlsbad, CA) in M2 medium for 20 min. H2DCFDA is a nonfluorescent chemical which can pass through the plasma membrane and converts to green fluorescent upon oxidation with ROS [56]. Oocytes were washed three times in H2DCFDA-free media before imaging using Leica SP5 spectral scanning confocal microscope. ImageJ software was used to quantify fluorescence.

### Quantification of mtDNA copy number in oocytes

To quantify mtDNA levels in individual oocytes, *Cox3* fragment was amplified using the primers shown in Supplementary Table 1 and subcloned into pCR™2.1-TOPO® - cloning vector (Invitrogen, Carlsbad, CA) as previously described [57]. One Shot TOP10 Chemically Competent E. coli were transformed and grown overnight at 37ºC. Recombinant plasmids were purified using Qiagen plasmid isolation kit and the inserted mtDNA fragment was confirmed by DNA sequence analysis. Plasmid DNA was quantified using NanoDrop 2000 spectrophotometer (Thermo Scientific, Waltham, MA). A standard curve from 10^8^ to 10^1^ plasmid molecules was generated by serial 10-fold dilutions. Single oocytes from *Mfn2^-/-^* and WT mice were individually lysed in 10 μl lysis solution containing 125 μg/ml Proteinase K and 17 μM SDS in sterile water by incubating at 55ºC for 2 h. Then, proteinase K was inactivated by heating the lysis mix at 95ºC for 10 min and the mix was used directly for downstream PCR. Reactions were performed in triplicates. Each 10 μl reaction contained 5 μl of SYBR Green supermix (Bio-Rad Laboratories, Hercules, CA), approximately 0.3 μM of each primer, and 1/3 of oocyte’s total DNA. Each individual oocyte’s mtDNA copy number was extrapolated from the standard curve.

### Immunostaining of oocyte spindles

To stain the spindles, oocytes were fixed in 4% (w/v) paraformaldehyde in DPBS (pH 7.4) for 30 min, and permeabilized in 0.5% Triton X-100 for 5 min. Then, oocytes were incubated with 2 μg/ml anti-α-Tubulin (Millipore, Billerica, MA) for 1 h, washed three times for 5 min in DPBS and stained with 4’,6-diamidino-2-phenylindole (DAPI, Life Technologies, ThermoFisher Scientific, Waltham, MA) prior to being examined using Leica SP5 spectral scanning confocal microscope (Leica Microsystem, Buffalo Grove, IL) using excitation at 488 nm and emission at 530 nm (α-Tubulin), and excitation at 350 nm and emission at 470 nm (DAPI).

### Staining of transzonal processes

Cumulus oocyte complexes (COCs) and secondary follicle enclosed oocytes (SFOs) were collected and fixed in 2% formaldehyde (Sigma, St. Louis, MO) for 1 h at 37°C, then washed in phosphate buffered saline with 0.1% Triton X-100 and 0.01% PVA and blocked with 3% BSA for 30 min at room temperature. Rhodamine-phalloidin (Life Technologies, Carlsbad, CA) diluted 1:40 in PBS/PVA was used to label the F-actin TZPs. In the last step, follicles were washed three times in PBS/PVA. Then oocytes were imaged by Leica SP5 spectral scanning confocal microscopy.

### Immunofluorescence staining

To perform immunofluorescence staining on paraffin embedded tissues, paraffin wax was removed by heating the slides for 45 min at 65°C. Tissue sections were then rehydrated with xylene and 100% ethanol. To retrieve antigens, slides were incubated with sodium citrate buffer (10 mM, pH 6.0) in a pressure cooker for 1 h. Slides were then cooled down to 27°C, and treated with 0.5% Triton X-100 for 10 min to permeabilize the tissue. To block the non-specific binding, slides were incubated in 2% Bovine Serum Albumin (BSA) for 45 min. Following the washing steps, slides were incubated overnight at 4°C with primary antibodies for E-cadherin, Connexin 37, Caspase-6 (Santa Cruz Biotechnology, Dallas, TX) or Ceramide (Enzo Life Sciences, Farmingdale, NY) diluted 1:50. The next day, slides were washed in 0.5% BSA diluted in PBS and incubated for 60 min at room temperature with Alexa Fluor 594-conjugated or Alexa Fluor 488-conjugated secondary antibodies (Thermo Fisher Scientific, Waltham, MA) diluted 1:200. Slides were then incubated with 4’, 6-diamidino-2-phenylindole (DAPI;1:1000) (Life Technologies, Carlsbad, CA) and stored at 4°C until imaging.

To perform immunofluorescent staining on oocytes, they were collected and fixed with 4% paraformaldehyde (Sigma, St. Louis, MO) in DPBS for 5 min, placed in 0.5% Triton X-100 in DPBS at room temperature for 30 min, followed by three times wash in DPBS. After blocking in 3% BSA (Sigma, St. Louis, MO) at room temperature for 1h, oocytes were incubated overnight at 4°C with mouse anti-TRF1 monoclonal antibody (Abcam, Cambridge, UK) and rabbit anti-53BP1 monoclonal antibody (Cell Signaling Technology, Danvers, MA). After three washes with DPBS, oocytes were incubated with Alexa Fluor 594-conjugated goat anti-rabbit antibody and Alexa Fluor 488-conjugated goat anti-mouse antibody for 1h at room temperature. For Mito-tracker and ER-tracker staining, oocytes were collected, fixed and then labeled with MitoTracker Red CMXRos (Invitrogen, Carlsbad, CA) and ER-Tracker Blue (Invitrogen, Carlsbad, CA) for 30 min at 37°C, washed with DPBS and mounted on glass slides. Images were captured on Leica SP5 spectral scanning confocal microscope and Image J software was used to quantify the fluorescence intensity.

### AMH and FSH testing

To measure the serum levels of Anti-Mullerian hormone (AMH) and follicle stimulating hormone (FSH), samples were sent to University of Virginia Center for Research in Reproduction Ligand Assay and Analysis Core, where ANSH ELISA kit was used to measure AMH levels, in-house RIA (radioimmunoassay) was used to measure FSH.

### RNA sequencing and data analysis

Five secondary follicle enclosed oocytes and GV oocytes each collected from *Mfn2*^-/-^ or WT mice (n=3) were pooled, respectively, and cDNA was amplified using Smart-Seq2 protocol as previously described [58,59]. Briefly, oocytes were lysed and mRNA was captured and amplified by using Smart-seq2 v4 kit (Clontech). After pre-amplification and AMPure XP beads purification, amplified RNAs were quality checked using Agilent High Sensitivity D5000 kit (Agilent Technologies). RNA sequencing libraries were constructed using Nextera® XT DNA Library Preparation Kit (Illumina, San Diego, CA) and multiplexed using Nextera® XT Index Kit (Illumina, San Diego, CA). Libraries were quantified by Qubit and Tapestation 4200. Indexed libraries were then pooled and sequenced on Illumina’s Hiseq 2500 platform with 100 bp pair-end reads. In total, we analyzed 12 samples and obtained approximately 212 million reads. The raw FASTQ files and normalized read counts are available at Gene Expression Omnibus (GEO) (http://www.ncbi.nlm.nih.gov/geo) under the accession number (GSE128842).

For RNAseq data analysis, multiplexed sequencing reads that passed filters were trimmed to remove adapters using Cutadapt and low quality reads were pre-filtered by FASTX-Toolkit before mapping. Clean reads were aligned to the mouse genome (GRCm38/mm10) using STAR with default parameters. Individual mapped reads were quantified to annotation model to calculate gene counts. Differential expression analysis between *Mfn2*^-/-^ or WT groups was performed using Partek Flow GSA algorithm with default parameters. Differentially expressed genes between *Mfn2*^-/-^ and WT groups were determined using false discovery rate (FDR) of *p* value < 0.05, foldchange > 2 and a minimal of 5 reads as cutoffs. Cluster analyses were performed by the K-means clustering algorithm using R. DAVID and Ingenuity Pathway Analysis (IPA) software were used to perform Gene Ontology (GO) and pathways analyses, respectively.

### Telomere length measurement

Approximately 20 oocytes and their surrounding cumulus cells were collected respectively and stored at –80 ºC until use. DNA was extracted from oocytes and cumulus cells using QIAmp DNA micro Kit (Qiagen,Valencia, CA) and quantified. Approximately 0.5 ml blood sample were collected form each group of mice and blood DNA was extracted using DNA isolation kit for mammalian blood (Roche, Basel, Switzerland). Average telomere length was measured from total genomic DNA using a real-time PCR assay, as previously described [28]. Telomeric primers and primers for the control gene (mouse 36B4 single copy gene) are listed in Supplementary Table 1. For each PCR reaction, a standard curve was generated by serial dilutions of known amounts of DNA from the same tissues. The telomere signal was normalized to the signal from the single-copy gene to generate T/S ratio. The relative telomere length is indicated by T/S ratio.

### Statistical analysis

Quantitative data are expressed as mean ± SEM. Student’s t-test was used to analyze the statistical significance between two groups. Data are representative of at least three independent experiments unless otherwise specified. All statistical analyses were done using Graph Pad Prism software version 7 and significance was assessed at *p* < 0.05.

## SUPPLEMENTARY MATERIAL

Supplementary Figures

Supplementary Table
