# Supplementary material for: Mitofusin 2 plays a role in oocyte and follicle development, and is required to maintain ovarian follicular reserve during reproductive aging
Source: Aging (Albany NY). 2019 Jun 16;11(12):3919–38. doi: 10.18632/aging.102024 (PMC6628992; doi:10.18632/aging.102024)
Supplement: Supplementary Figures [file aging-11-102024-s002.pdf]

SUPPLEMENTARY MATERIAL

Supplementary Figures

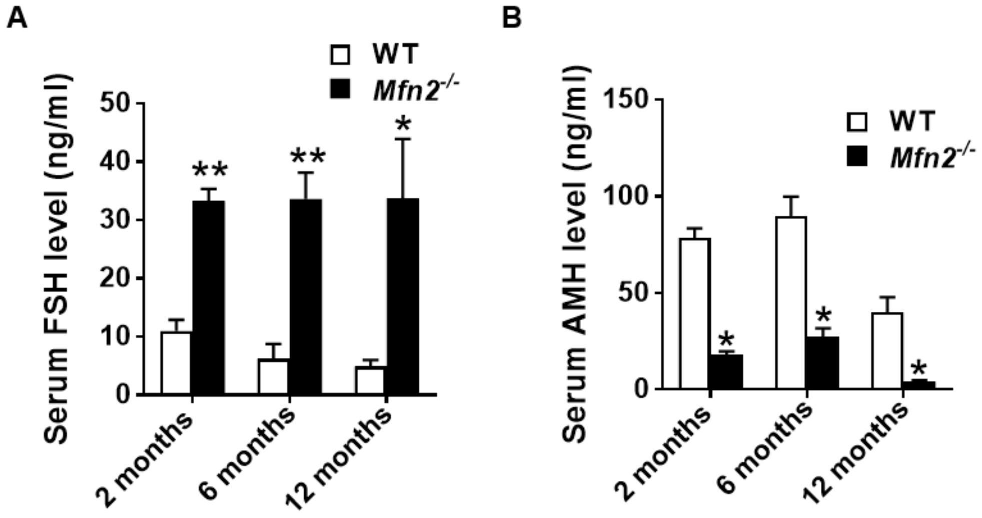

Supplementary Figure 1. Serum AMH and FSH levels in female *Mfn2*$^{-/-}$ mice. (A, B) Serum FSH and AMH levels in 2, 6- and 12 months old female WT and *Mfn2*$^{-/-}$ mice. Data presented as mean ± SEM. *$p < 0.05$, **$p < 0.01$ vs. WT from *t*-test.

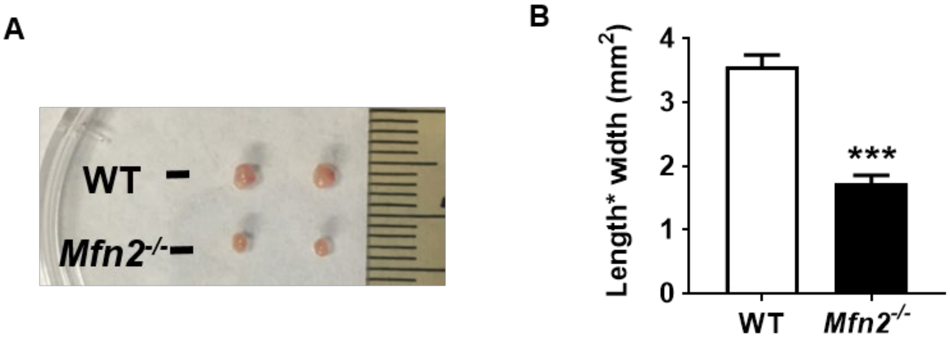

Supplementary Figure 2. Ovarian size is decreased in *Mfn2*$^{-/-}$ mice. (A) Representative photographs of ovaries from 8-week-old *Mfn2*$^{-/-}$ and WT mice. **(B)** *Mfn2*$^{-/-}$ mice had significantly smaller ovaries. Data presented as mean ± SEM. **$p < 0.01$ vs. WT from *t*-test.

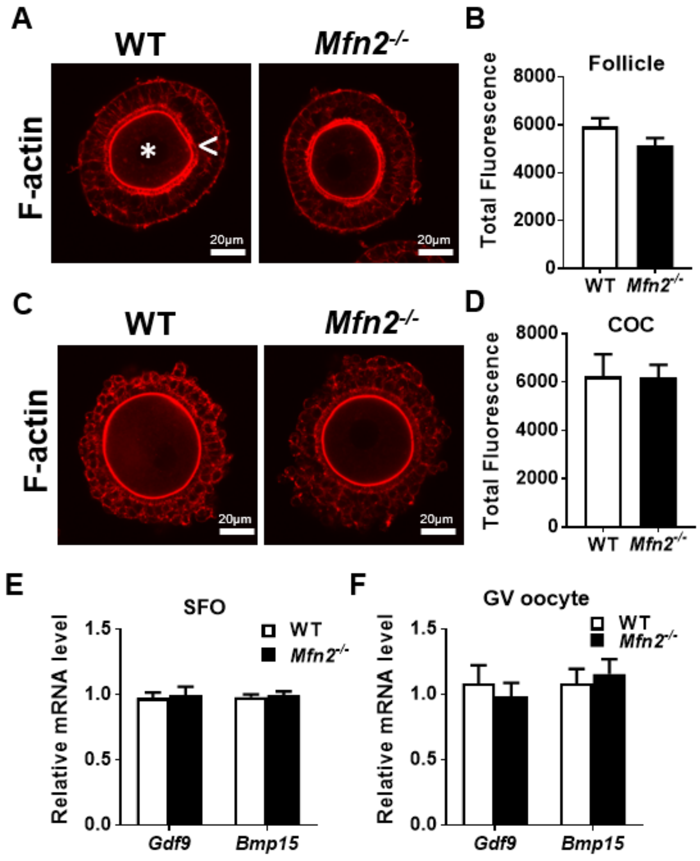

Supplementary Figure 3. TZP formation is not changed in *Mfn2*$^{-/-}$ secondary follicles of COCs. (A, C) Secondary follicles and COCs were isolated from the ovaries of *Mfn2*$^{-/-}$ and WT mice, fixed, labeled with rhodamine-phalloidin, and examined by confocal microscopy. Asterisk designates oocyte; open arrow designates labeling of TZPs. **(B, D)** The total peak intensity for the zona pellucida and plasma membrane was calculated. **(E, F)** qRT-PCR analysis of *Gdf9* and *Bmp15* expression in *Mfn2*$^{-/-}$ and WT SFOs and GV stage oocytes. Data presented as mean ± SEM.
