# Supplementary material for: Mitofusin 2 plays a role in oocyte and follicle development, and is required to maintain ovarian follicular reserve during reproductive aging
Source: Aging (Albany NY). 2019 Jun 16;11(12):3919–38. doi: 10.18632/aging.102024 (PMC6628992; doi:10.18632/aging.102024)
Supplement: Supplementary Table [file aging-11-102024-s001.pdf]

Supplementary Table

Table S1. The list of primers used for quantitative RT-PCR

Gene	TaqMan assay number or Primer sequences (5' to 3'; F, forward; R, reverse)
β-actin	Mm00607939_s1
β-actin	F: GGCTGTATTCCCCTCCATCG<br>R: CCAGTTGGTAACAATGCCATGT
Atp5a1	Mm00431960_m1
Bmp15	Mm00437797_m1
activin-β	F: AGATCCCGCACCTCGAC<br>G: TGGTTGCCTTCATTAGAGACG
Cox1	Mm04225243_g1
Cox3	F: TTTGCAGGATTCTTCTGAGC<br>R: TGAGCTCATGTAATTGAAACACC
Cre	F: ATGCTTCTGTCCGTTTGCC<br>R: CAACACCATTTTTTCTGACCC
Dnaja3	Mm00469723_m1
Gdf9	Mm00434083_m1
Hspd1	F: AGTGGATGACCTCGTGTTATGC<br>R: GGATCTAGTTTCTGGCCTCTTCG
Hspe1	F: TGGTAATCTTTAGCGGTGCTC<br>R: GGAGGACTTTATCCCACAGC
Mfn2 flox	F: GAAGTAGGCAGTCTCCATCG<br>R: AACATCGCTCAGCCTGAACC
Ndufv1	Mm00504941_m1
Sdhb	Mm00458272_m1
Uqcrc2	Mm00445961_m1
Telomeric	F: CGG TTT GTT TGG GTT TGG GTT TGG GTT TGG GTT TGG GTT<br>R: GGC TTG CCT TAC CCT TAC CCT TAC CCT TAC CCT TAC CCT
36B4 control	F: ACT GGT CTA GGA CCC GAG AAG<br>R: TCA ATG GTG CCT CTG GAG ATT

Abbreviations: *Atp5a1*: ATP synthase, H+ transporting, mitochondrial F1 complex, alpha subunit 1; *Bmp15*: bone morphogenetic protein 15; *Cox1*: cytochrome c oxidase subunit I; *Cox3*: cytochrome c oxidase subunit III; *Drp1:* Dynamin related protein 1; *Gdf9*: growth differentiation factor 9; *Mfn2*: mitofusin 1; *Ndufv1*: NADH dehydrogenase (ubiquinone) flavoprotein 1; *Opa1*: mitochondrial dynamin like GTPase; *Sdhb*: succinate dehydrogenase complex iron sulfur subunit B; *Uqcrc2*: ubiquinol cytochrome c reductase core protein 2.
